# Building Application-Related Patient Identifiers: What Solution for a European Country?

**DOI:** 10.1155/2008/678302

**Published:** 2008-03-31

**Authors:** Catherine Quantin, François-André Allaert, Paul Avillach, Maniane Fassa, Benoît Riandey, Gilles Trouessin, Olivier Cohen

**Affiliations:** ^1^Service de Biostatistique et Informatique Médicale, CHU de Dijon, INSERM EMI 0106, 21079 Dijon Cedex, France; ^2^Department of Epidemiology and Biostatistics, Mc Gill University, Montreal, Canada QC H3G1Y6; ^3^Laboratoire d'Epidémiologie, Statistique et Informatique Médicales (LESIM), Université Victor Segalen Bordeaux 2, 146 rue Léo-Saignat, 33076 Bordeaux Cedex, France; ^4^Laboratoire d'Enseignement et de Recherche sur le Traitement de l'Information Médicale, Faculté de Médecine, Université de la Méditerranée Marseille, 13284 Marseille Cedex 07, France; ^5^Institut National d'Etudes Démographiques (INED), 133 Boulevard Davout 75980 Paris Cedex 20, France; ^6^OPPIDA Sud, Batiment F 78 allée Jean Jaurès, 31000 Toulouse, France; ^7^HC Forum, Les Jardins de Maupertuis, 7 Chemin de la Dhuy, 38240 Meylan, France

## Abstract

We propose a method utilizing a derived social security number with the same reliability as the social security number. We show the anonymity techniques classically based on unidirectional hash functions (such as the secure hash algorithm (SHA-2) function that can guarantee the security, quality, and reliability of information if these techniques are applied to the Social Security Number). Hashing produces a strictly anonymous code that is always the same for a given individual, and thus enables patient data to be linked. Different solutions are developed and proposed in this article. Hashing the social security number will make it possible to link the information in the personal medical file to other national health information sources with the aim of completing or validating the personal medical record or conducting epidemiological and clinical research. This data linkage would meet the anonymous data requirements of the European directive on data protection.

## 1. INTRODUCTION

In the majority of industrialized countries, patient identification lies at the heart of many of the concerns relating to electronic processing of health information. In August 2004, the French government decided by law to start a national project for an electronic health record called the “dossier médical personnel,” the personal medical record (PMR) [1]. It is designed to promote health care coordination, enhance communication of health information, and reduce iatrogenic accidents. The information system corresponding to this project is still under construction. Regarding the patient identifier, one of the proposals has been to use the existing social security number (SSN).

However, civil society and bodies representing citizens (patients' associations, those defending individual liberties, national councils of medical associations) are worried, quite rightly, about the security of medical information [2] should the SSN be used as an identifier in the field of health. They are afraid that medical information may be linked with other information (social, economic, financial, employment) often identified by the SSN. This is one of the reasons why the government decided to postpone this project (November 2007) [3]. Nevertheless, in December 2007, a working group was mandated by the government to propose, as soon as possible, a solution for the HIN, which will be used not only for the DMP project but also for health in general.

Through this paper, we first propose a method which aims both to reassure citizens' representatives regarding the security of medical information and to give the opportunity to the French government to utilize a derived SSN with the same reliability as the SSN. A solution based on the utilization of anonymity techniques is proposed.

Secondly, we analyse the conditions necessary to make this first solution interoperable at the European level.

## 2. BACKGROUND

In an increasing number of countries, patients have direct access to their medical records (MR), and some countries including France [1] have decided to provide a personal medical record (PMR) to the patient. We define MR [4] as patients' medical information recorded by the medical practitioner under his or her own responsibility and ideally electronically signed by him/her in order to authenticate the provider (health professional) and to prevent any modification of its content.

In contrast, the PMR is personally supervised by each patient, who has the right to mask any information he/she does not want to be read. As the owner of this PMR, the patient determines who can gain access to his/her record. Of course, patients are very worried that information concerning their private lives may be disclosed. Such concerns are increasing as more sensitive medical details, such as psychiatric records, HIV status, and genetic information, are stored in their PMRs. In France, to enhance the coordination of care, the PMR's data will be stored in national shelters. So patients need to be sure that medical information may not be linked with other information (social, economic, financial, employment) also identified by the SSN, if the SSN was used as an identifier for the PMR.

Many other countries have chosen a homogenous national patient identification system (e.g., Northern European countries such as Denmark, Finland, Luxembourg, the Netherlands, Belgium, the United Kingdom, and Ireland) [5], and the former countries in this list are even using the same identification number for other fields than health care. Apart from Belgium, the above-mentioned countries implemented a unique patient identifier some time ago, and the citizens are used to it and to the creation of national health databases. However, in the United Kingdom, patients are reassured because there is no national shelterer and medical records are stored by the general practitioner who cannot transmit or receive any information regarding a patient without his/her consent.

In Southern European countries identification is often organized at a regional level (Spain and Italy) and patients are more familiar with the creation of large health databases organized at a federal level as in other countries (Canada and the USA).

To reassure French patients regarding the security of their medical data which will be stored at a national level, we propose in this paper the creation of a secure patient identifier which should be different from the SSN to avoid linkage with other data, but which should be as reliable as the SSN. This proposed solution could be used by any country needs to implement a unique health identification number.

## 3. MATERIALS AND METHODS

### 3.1. A solution to the security problems

As we said before, it is perfectly possible to preserve the confidentiality expected by the patient by putting in place anonymity procedures [6, 7] such as those adopted by the Institut de Veille Sanitaire (the French Institute for Public Health Surveillance) [8] on the recommendation of the French National Commission for Data protection, in the context of followup procedures for the 30 diseases subject to mandatory reporting (including AIDS).

Unlike encryption methods that must be reversible to allow the legitimate recipient to decode the message, unidirectional hashing techniques, such as the standard hash algorithm in its modified version SHA-2 (SHA-2 is considered “significantly stronger” than SHA-1, although somewhat slower: NSA and NATO recommend it in the SUITE-B package (ECC, AES and SHA-2.)), are irreversible. Hashing produces a strictly anonymous code (it is not possible to retrace the patient identity) that is always the same for a given individual and thus ensures that patient data can be linked. There are many medical applications including the creation of national databases (such as those relating to the national followup of infected persons—approximately 100 000 patients—which are an excellent example of how it can help epidemiological research [9], with complete patient approval [10]) as well as regional and interregional databases in many areas (cancer, perinatal diseases, genetic diseases). This system, which is based on the hash coding of the social security number [11], the gender and the date of birth, has also made it possible to link all standardized hospital discharge abstracts, classified into French diagnosis-related groups at the French national level, and to link the data of the national medical insurance information system. An anonymity procedure based on hash coding is also used to chain patient files in Switzerland [12]. Similar solutions [13], also derived from the irreversible encryption of the unique social security number, have been proposed in Belgium [14] and New Zealand.

In the case of the PMR, the situation is no more complex because similar requirements must be met.

- The French Commission for data protection, and associations of patients and healthcare professionals demand the confidentiality of personal information contained in the PMR.
- Public health bodies or individuals need to have access to these data, particularly when the patient has given express consent.

Ideally, hashing the social security number would help to meet these requirements. Regarding confidentiality, insofar as the social security number (SSN) could not be reconstituted using the health identifier number (HIN), the link between them would be broken. Another advantage of using hash coding is that it meets the criterion of being focused (created and maintained solely as a support for health care). This is one of the main criteria of a universal health care identifier that were published by the American Society for Testing and Materials, a standards development organization accredited by the American National Standards Institute, in the standard guide for properties of a universal healthcare identifier [15].

This solution satisfies the demands of French Commission for data protection in that the SSN would be rendered anonymous by a trustworthy third party and would thus permit the generation of a health identifier number (HIN) known by the patient. This supposes that the HIN would be included in the patient smart card, used for administrative and financial purposes (reimbursement of medical care).

For French patients associations, the interest and importance of printing the health identifier number (HIN) on the patient smart card lie in the fact that it would mobilize and sensitize patients, and increase their awareness that the identifier is different from the SSN. In fact, if the SSN was rendered anonymous using a hash algorithm, there would be no link between the new number and the personal details of the individual, which is not the case for the SSN. Nevertheless, it seems to us that this solution is an illusion in terms of security and reliability because the correspondence between the SSN and HIN would be set out on the patient smart card and would therefore also be known by the health workforce: the SSN is needed in the smart card for reimbursement purposes.

**Figure 1:**
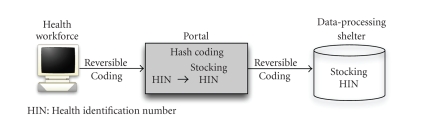
Schema of the proposed solution.

With regard to methodology, to provide anonymity safely requires double hashing, because anonymity is not guaranteed by a single hashing process [6, 7]; two hashing keys must be used to obtain complete anonymity. As a consequence, this solution must be completed by the following principles. According to Figure 1, the health professional can provide to the portal of the application a coding HIN obtained through reversible encryption. The HIN hashing transformation is carried out by the portal. The HIN will itself be transformed into a stocking HIN in the same way that the SSN was transformed into an HIN, but with a different key (see Figure 1). This portal can then transmit this code to the data-processing shelterer safely and reliably.

If a person manages to get the SSN of a patient and uses the hash function, he has to know the key used by the trustworthy third party to obtain the HIN of the patient. This is very difficult. However, as the SSN and the HIN of the patient will both be included in the smart card of the patient and used by health professionals, there is a risk of a breach of privacy in health structures. That is why we propose to apply a double-hash procedure. The aim of hash coding of the HIN by the Portal (with a more different key than that used for hash coding the SSN) is to obtain an anonymised identifier (stocking HIN) before sending it to the data processing shelter.

It also seems fundamental to us that prerequired conditions be imposed in terms of security and confidentiality [18] regarding access to medical data in health structures. This raises several concerns, the first of which is related to the authentication of health-care professionals. The solution would be a smart card attributed to professionals in both the private sector and public hospitals. In France, the use of such cards was imposed in May 2007. Our second concern is the archiving of health data by health professionals. It is not only about controlling the right of access to different applications, but also about ensuring the security of local hard disk storage, preventing direct access to the databases, and guaranteeing the security of external archives (Band and CD-ROM). Lastly, we are also very concerned about the security of exchanges among health professionals. We must point out that many health-care professionals require access to medical records from home, for example, or when on call. A similar situation arises when records are forwarded to doctors during vacations through the internet. We also know that some data processing specialists or computer science experts need access to health structure data for application maintenance. We then strongly recommend the use of networks like virtual private network, in addition to the professional authentication card, as a solution to the problem of the security of data exchange.

### 3.2. Conditions for making this solution interoperable

Mobility must not compromise healthcare for European nationals. Residents of one member state travelling to or working in another member state should have the same right to high-quality healthcare as all other Europeans (see, e.g., Regulation (EC) No 883/2004). It is thus very important to ensure interoperability between the patients identifiers in the different European countries. However, in Europe, each country has its own patient identifier with very different structures and contents, and which may not always be derived from an SSN. As a consequence, the use of the SSN alone (or a derived SSN) to generate the HIN in European countries would not solve the interoperability problem.

One solution would be to add personal patient characteristics such as family name, first name, date of birth (separately hashed) to be able to link data even in the case of field errors or other local errors to the SSN, which would help to comply with the recommendations of the International Association of Medical Information Technology, and where possible to ensure interoperability of this identifier with a European identifier [16].

We propose to add to the European health card, as well as the national social insurance number of the patients in each country, a family-based identifier which could contribute to the harmonisation of patients' identification at the European level. This solution would lead to the creation of a European health identifier which would allow patient data to be gathered anywhere in Europe, whatever their location, even if their social insurance number changes according to their country of residence. This will be useful, at the individual level, to provide higher quality health care due to a better followup of the patient and to facilitate the reimbursement of health care costs. At the community level, this will increase the reliability of public health statistics. The great advantage of this European health identifier based on the family component is that it is built from very basic information, which is available to everybody, easily checkable and permanent throughout the patient life. This is not the case for the social insurance number. Our proposal to use the family-based identifier to create a unique European identifier will make it possible to link the data of a patient even when he or she resides outside his or her home country in Europe. It will also contribute to the establishment of European public health statistics by matching healthcare data of the patients records with other administrative data (mortality, social information, etc.) after anonymisation of these data in accordance with the European directive on data protection.

We could also propose to use biometric technologies (and then apply hash coding to ensure anonymity). Biometric technologies are sometimes proposed as a way to associate a patient with his or her medical data, as they do not require the patient to bring any documents or remember any information. Though this technology represents real progress both in the identification and in the authentication of the patient, there are still many questions [17] regarding the accuracy and reliability of each biometric technology and the associated costs. But the main problem lies in the acceptability of such systems by organisations concerned with ethical considerations such as patients' associations, national ethics committees, human rights associations, and national committees for data protection. For example, in France, the use of biometric solutions for identification in the field of health has not been approved by the National Ethics Committee.

## 4. DISCUSSION

Initially, the government planned to use the SSN as an HIN, as health professionals already know the SSN for health reimbursement reasons. Patients' associations, those defending individual liberties, national councils of medical associations, have asked for an identity number HIN that is different from the SSN, their requirements are the following.

- First, that patients are made aware that the HIN used for medical care is not the same as the SSN.
- Second, that there are two different databases for health professionals (located in hospitals or in other structures): a database for health reimbursements which will exclusively use the SSN for administrative reasons, and another medical care database for medical purposes which will not use the SSN because of its private aspect, but an anonymous identity number HIN different from the SSN.

We had to comply with these requirements and are thus obliged to provide tables to show the link between the SSN and the HIN for all patients in all health structures for administrative purposes, which can be considered as the most important disadvantage of the proposed solution.

Patients' associations and national councils of medical associations have been aware of the need for a correspondence table between administrative databases (for health reimbursements) and personal databases (for medical purposes). This correspondence table was not a real problem for them as such tables currently exist in hospitals because of the need to link medical personal data (hospital discharge abstracts referenced by the first and second name, date of birth, etc.) with billing data referenced by the SSN. This particularly arises from the fact that administrators need to have access to medical information in order to determine the levels of hospital activity and justify hospital budgets. The solution for the existing correspondence table between administrative databases (for health reimbursements) and personal databases (for medical purposes) was to put it under the responsibility of the medical information department. That is why the associations concerned consider that using an HIN obtained through irreversible transformation of the SSN provides more security than the current situation where the medical data are stored with personal characteristics (first and second name, date of birth, etc.).

We consider that our proposal must be accompanied by the reinforcement of measures regarding the security of medical information inside hospitals, as proposed at the end of the Section 3. In particular, we focused on the need to code (reversible encryption) both the HIN and SSN in hospitals when they are related to health-care databases.

As a consequence, the main concern regarding the personal medical record (PMR) and centralised storage of patient medical information is the security of data, not only at the level of the health structures, but also at the data-processing shelterer. Knowing that a correspondence table between the SSN and the first HIN would exist in health structures, we propose transforming the first HIN into a storing HIN by using an irreversible encryption method to secure storage (data-processing shelterer). As only the manager of the portal will know the key of this second hashing, no dictionary attacks can be used to try to obtain the first HIN, and thus the corresponding SSN through the correspondence table (if hashed at the health structure level). 

Concerning the proposed solution to security problems, this method has several advantages. First, in terms of organisation, it avoids implementation of the hashing function at the level of the health professional. Second, it also avoids supplementary costs, implementation delays, and the disclosure of the technical hashing system to other actors.

The second advantage is that it provides the possibility to use different hash-coding keys to generate a different HIN for each major application such as the PMR, regional, and/or national applications such as health care networks, health administration, diagnosis and healthcare, and epidemiological research without delivering the corresponding application keys to all of the health professionals. Moreover, hashing responsibility and key management can be delegated to each application. 

If the concept of specific HINs for different applications is accepted, it will be necessary to create a structure similar to the Data Matching Agency created in Australia to manage all of the identifiers. This agency could be in charge of matching with regard to legal, deontological, organisational, and technical aspects, since the trustworthiness of such an agency is based on a complex combination of legal obligations (in order to provide justifiable legal reliability) and organisational-and-technical security measures (in order to provide justifiable robustness with regard to accuracy of data).

Management of HINs consists in not only ensuring the linkage of the different databases, but also guaranteeing the security of the different identifiers. For example, the real risks raised by using the same identifier in regional and national applications have to be considered: it is important to preclude the possibility of unauthorized linkage by indirect means. This agency could also manage the distribution of encryption keys among the different applications and actors. To ensure that legislation is respected and the interests of the patients protected, the agency would liaise with national commissions for data protection.

## 5. CONCLUSION

Our proposal for a French health identification number would make it possible to uniquely identify and link a patient to his or her specific medical data. Hashing the social security number will allow linkage between the information of the personal medical file and other national sources of health information with the aim of completing or validating the personal medical record or performing epidemiological research.

Adding personal patient characteristics such as first name and family name, date of birth and/or biometric identifiers (separately hashed, then merged) to the hashed social security number could also be proposed in the discussions about the creation of a European health care identifier. It would thus contribute to the establishment of European public health statistics by matching healthcare data of the patients records with other administrative data. These data linkage systems would meet the requirements for anonymous data issued in the European directive on data protection.
